# Interaction between hexon and L4-100K determines virus rescue and growth of hexon-chimeric recombinant Ad5 vectors

**DOI:** 10.1038/srep22464

**Published:** 2016-03-03

**Authors:** Jingyi Yan, Jianing Dong, Jiaxin Wu, Rui Zhu, Zhen Wang, Baoming Wang, Lizheng Wang, Zixuan Wang, Haihong Zhang, Hui Wu, Bin Yu, Wei Kong, Xianghui Yu

**Affiliations:** 1 National Engineering Laboratory for AIDS Vaccine, School of Life Sciences, Jilin University, Changchun, Jilin, China

## Abstract

The immunogenicity of recombinant adenovirus serotype 5 (rAd5) vectors has been shown to be suppressed by neutralizing antibodies (NAbs) directed primarily against hexon hypervariable regions (HVRs). Preexisting immunity can be circumvented by replacing HVRs of rAd5 hexon with those derived from alternate adenovirus serotypes. However, chimeric modification of rAd5 hexon HVRs tends to cause low packaging efficiency or low proliferation of rAd5 vectors, but the related mechanism remains unclear. In this study, several Ad5-based vectors with precise replacement of HVRs with those derived from Ad37 and Ad43 were generated. We first observed that a HVR-exchanged rAd5 vector displayed a higher efficacy of the recombinant virus rescue and growth improvement compared with the rAd5 vector, although most hexon-chimeric rAd5 vectors constructed by us and other groups have proven to be nonviable or growth defective. We therefore evaluated the structural stability of the chimeric hexons and their interactions with the L4-100K chaperone. We showed that the viability of hexon-chimeric Ad5 vectors was not attributed to the structural stability of the chimeric hexon, but rather to the hexon maturation which was assisted by L4-100K. Our results suggested that the intricate interaction between hexon and L4-100K would determine the virus rescue and proliferation efficiency of hexon-chimeric rAd5 vectors.

Recombinant adenoviruses have attracted tremendous interest as gene delivery vectors due to their ability to efficiently infect a variety of cells and to be generated to high titers *in vitro*^1^,^2^,^3^,^4^. Because of these and other features, many studies focus on the construction of novel genetically capsid-modified adenovirus vectors using genetic engineering for gene therapy and vaccine at present. Hexon, which is the main capsid protein, is an important target for the genetic modification of adenovirus vectors. Currently, the strategies of genetic modification on hexon include: chimeric modification to circumvent preexisting immunity^5^, antigen capsid-incorporation to enhance immunogenicity and protection of vaccines^6^, targeting modification for cancer gene therapy^1^. However, genetic modification on hexon tends to cause low packaging efficiency or low proliferative capacity of adenovirus vectors, and herein the related mechanism is not yet clear. Therefore, clarifying the assembly mechanism of genetically hexon-modified adenovirus vectors, and help to establish a high throughput platform or method to evaluate the packaging capability of genetically modified adenovirus vectors, so as to provide guidance and help for the design and construction of novel adenovirus vectors for vaccines and gene therapy. As hexon-modified adenovirus vectors now focus on the HVRs which is on the hexon surface, we believed that studies based on hexon- chimeric Ad5 vectors will help understand other types of hexon-modified adenovirus vectors.

At least 12 proteins contribute to the capsid structure of the virion, and several additional proteins participate temporarily in the assembly process^7^,^8^. Prior studies suggested that the inability to rescue chimeric Ad5 vectors possibly may be due to a lack of structural compatibility between the alternate hexon proteins and cognate proteins during the process of virus assembly, such as the late phase L4-100K non-structural protein, which is involved in hexon assembly and transport to the nucleus or other adenovirus capsid proteins that interact with hexon in the final capsid^9^,^10^. The intricate folding and remarkable stability of the hexon trimer has been extensively described^11^,^12^,^13^,^14^,^15^,^16^. Evidence has been provided suggesting that the trimeric hexon is transported to the cell nucleus in the presence of the L4-100K protein and independently of any other adenovirus proteins. In addition, the L4-100K protein was found to interact with both hexon monomers and trimers within the cytoplasm, whereas it interacts predominantly with hexon trimers in the nucleus^17^,^18^. Based on these prior observations, we hypothesized that the L4-100K protein can bind chimeric hexon monomers but fail to assist in the proper folding of hexon trimers, thereby causing some hexon-chimeric adenoviruses to fail to rescue virus and others to grow efficiently to high titers.

Our previous studies revealed that complete modification of HVR1–7 resulted in a radical change in the antigenic specificity. Native HVRs proteins could recognize the serotype specific NAbs, and HVR-5 and 7 contained the Ad-specific NAb epitopes but not the key epitopes^18^. In this study, we replaced the hexon HVRs 5 and 7 or 1–7 of Ad5 with the equivalent HVRs from Ad37 and Ad43, both of which are subgroup D serotype viruses with low prevalence of NAbs in humans. We found that the Ad5-37(1–7) vector was nonviable. By contrast, Ad5-37(5, 7) and Ad5-43(1–7) vectors were viable but showed lower proliferation, similar to previous reports^5^,^19^. Unexpectedly, the Ad5-43(5, 7) vector showed higher efficacy of the recombinant virus rescue and could be produced to higher titers than the Ad5 vector. In this report, we consequently analyzed the structural stability of chimeric hexon proteins and their interactions with L4-100K proteins in order to determine the reason for the failure to be rescued and change in growth of hexon-chimeric rAd5 vectors.

## Results

### Variable recombination efficiencies of HVR-chimeric Ad5 vectors

**Figure 1:**
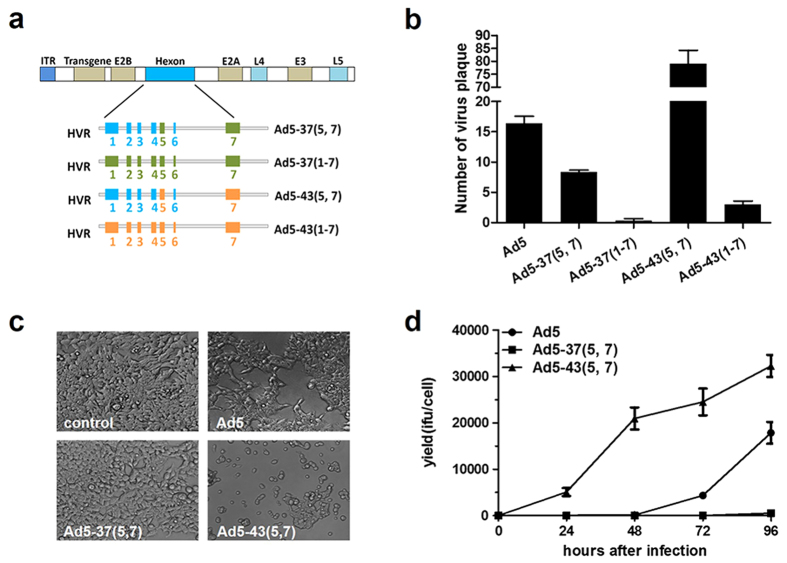
Construction of chimeric rAd5 vectors containing hexon HVRs from Ad37 and Ad43. Recombinants selected by GFP expression were identified by PCR, and their recombination efficiencies were calculated. (**a**) A schematic strategy to construct Ad5-37(5, 7), Ad5-37(1–7), Ad5-43(5, 7) and Ad5-43(1–7) vectors by exchanging either the fifth and seventh HVR or all seven HVRs with the corresponding regions. The Ad5 genes with the hexon protein, indicated in blue, are shown above. HVR sequences from Ad37 are indicated in green and Ad43 in orange. (**b**) The HVR-chimeric Ad5 genome plasmids together with the shuttle plasmid expressing GFP were co-transfected into HEK293 cells (1 × 10^6^ cells in 10 cm^2^ dish), single cytopathic effect (CPE) plaques expressing GFP were counted 10 days after co-transfection. (**c**) HEK293 cells were infected with the indicated virus at an MOI of 10 IFU/cell, and CPE images were taken 48 h after infection with the indicated chimeric adenovirus. (**d**) HEK293 cells were infected with the indicated virus at an MOI of 10 IFU/cell, chimeric adenoviruses were harvested at the indicated time points from cells by three freeze-thaw cycles, and vector yield was determined using rapid titer method. Data are means ± SD (n = 3).

In our previous study^18^, we showed that native HVR proteins could recognize serotype specific NAbs, and HVR5, 7 contained Ad-specific NAb epitopes. Thus, completely modifying HVR1–7 would result in a radical change in the antigenic specificity. To determine whether HVR5, 7 and HVR1–7 chimeric vectors can be successfully rescued, here, we replaced the hexon HVRs 5, 7 or 1–7 of Ad5 with the equivalent HVRs from Ad37 or Ad43 (Fig. 1a and supplementary Table 1), both of which are subgroup D serotypes with low prevalence of NAbs in humans. After constructing the chimeric genomic plasmids as described in materials and methods, an improved two-plasmid rescue method was performed to generate the chimeric Ad5 vectors and evaluate their recombination efficiencies. HEK293 cells were co-transfected with various combinations of Ad5 genomic plasmids and shuttle plasmids expressing green fluorescent protein (GFP). Green fluorescent cytopathic effect (G-CPE) plaques of Ad5 vectors were observed at indicated time points to 14 days post co-transfection (Fig. S1). We tested the recombination efficiency of these HVR-chimeric vectors by counting the G-CPE plaques 10 days after co-transfection, and the rAd5 vectors were identified by randomly picking single CPE plaques and confirming by PCR using E1, E2B and HVRs primers (Fig. S2). As shown in Fig. 1b, no positive plaques were detected in Ad5-37(1–7), and only two to four G-CPE plaques were observed in Ad5-43(1–7). Ad5-37(5, 7) was viable, although final yields were only about eight G-CPE plaques. Interestingly, Ad5-43(5, 7) generated the highest number of G-CPE plaques, which was nearly 6-fold greater than that of Ad5. To determine whether viral replication was altered, we assayed the production of Ad5, Ad5-37(5, 7) and Ad5-43(5, 7) in HEK293 cells. Afterwards, the infectious particles/physical particles (IFU/PP) of the HVR-chimeric rAd5 vectors were detected (supplementary Table 3). These vector yields were determined at indicated time points after infection at the multiplicity of infection (MOI) of 10 infectious titers (IFU)/cell, and observations of the cell morphology showed that viral CPE varied markedly between Ad5-37(5, 7) and Ad5-43 (5, 7) after 48 h post infection (h p.i.) (Fig. 1c). Virus yields were detected at 24, 48, 72 and 96 h p.i. (Fig. 1d). Genomic replication as quantified by q-PCR (Fig. S3), and late adenovirus proteins were also tested (Fig. S4). For both Ad5-37(5, 7) and Ad5-43(5, 7), the level of genome replication and the expression of late adenovirus proteins were similar to that of Ad5 at all time points^20^. However, productivity of these vectors showed remarkable differences at all times compared to Ad5. Ad5-37(5, 7) presented growth defects in viral progeny production relative to the Ad5 vector. By contrast, Ad5-43(5, 7) displayed a statistically significant 100-fold growth increase compared to the Ad5 vector (Fig. 1d). Since all of these chimeric vectors were identical except for the hexon sequences and showed similar viral DNA replication, these results suggested that the growth defect of Ad5-37(5, 7) may be due to alterations in hexon expression or capsid assembly.

### Differences in chimeric hexon protein expression and ability to properly fold into trimers

**Figure 2:**
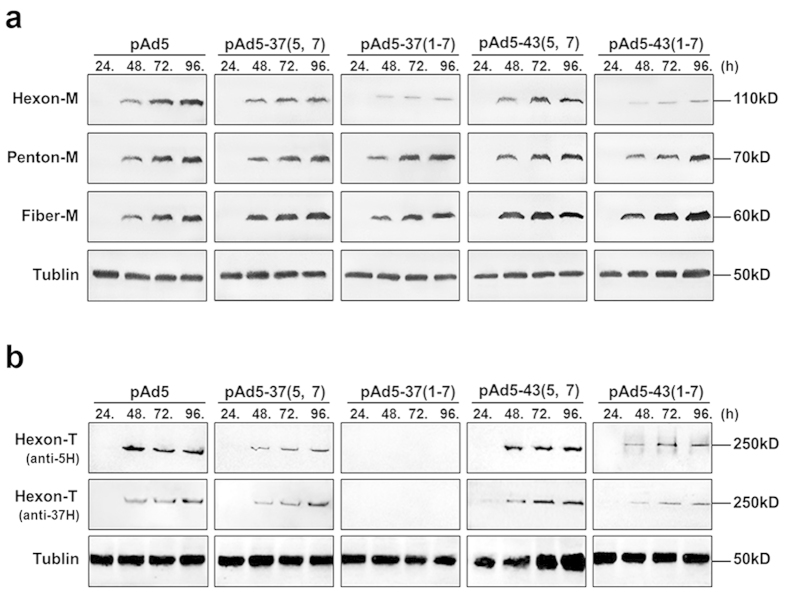
Detection of major capsid proteins. HEK293 cells were transfected with pAd5, pAd5-37(5, 7), pAd5-43(5, 7) pAd5-37(1–7) and pAd5-43(1–7) genomic plasmids. Cells were harvested at the indicated time points. (**a**) Total cell extracts were prepared, separated by SDS-PAGE and subjected to immunoblotting using anti-penton, anti-fiber or anti-hexon rabbit polyclonal sera and mouse tubulin mAb as a loading control. (**b**) Total-cell extracts were prepared, and hexon trimers were separated by native-PAGE and subjected to immunoblotting using anti-hexon goat pAbs from serotype 5 and serotype 37, and a mouse tubulin mAb was used as a loading control. All Western blotting experiments were repeated three times.

Due to some of chimeric Ad5 vectors failed to rescue viruses or produced poorly growing viruses, capsid protein expression were tested firstly by transfection of chimeric genomic plasmids, expressing most of adenovirus structural and non-structural proteins, could basically reflect the natural conditions. Western blotting was performed with an anti-Ad5 rabbit polyclonal antibody to test the protein expression of genomic plasmids pAd5, pAd5-37(5, 7), pAd5-37(1–7), pAd5-43(5, 7) and pAd5-43(1–7) by transfection of HEK293 cells. Cell lysates were prepared at indicated time points (Fig. 2a). Fortuitously, very few of the monomeric hexons in pAd5-37(1–7) were detected even after 96 hours post transfection. A similar difference was apparent in pAd5-43(1–7), which was less inclined to genetic recombination than pAd5, pAd5-37(5, 7) and pAd5-43(5, 7). By contrast, penton and fiber were expressed at similar levels from 48 h after transfection compared to the corresponding proteins in the control Ad5. These data clearly indicated that different yields for these structural proteins between chimeric adenovirus vectors were caused by hexon replacement. Furthermore, the capability of antibodies to hexon from serotype 5 to recognize hexon sequences from different subgroups should be considered. Thus, anti-hexon goat polyclonal antibodies (pAbs) for serotype 5 and serotype 37 were used to detect hexon monomers, but no additional chimeric hexon monomers were observed in pAd5-37(1–7) and pAd5-43(1–7) (data not shown). As each hexon capsomere is a homotrimer in the mature virion, we analyzed the trimeric hexon in non-denaturing conditions as shown in Fig. 2b. Western blotting showed that the abilities of the different anti-hexon antibodies to recognize different serotypes were similar. Intriguingly, hexons from pAd5-43(5, 7) were capable of forming trimers with high-efficiency, but those of pAd5-37(5, 7) and pAd5-43(1–7) were far less able to do so compared with wild-type Ad5. Notably, trimeric hexon from pAd5-37(1–7) was not detected. These findings implied that whether chimeric vectors were successfully rescued depended on the proper folding of hexon in which HVRs were replaced.

### Structural stability does not affect assembly of HVR-chimeric hexons

**Figure 3:**
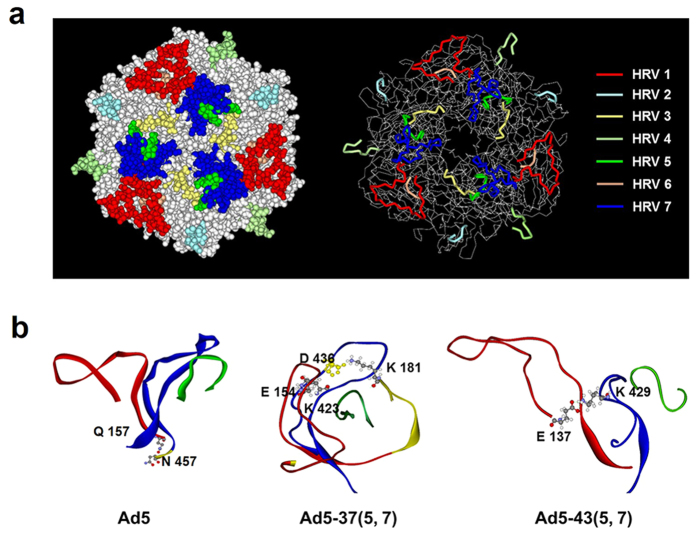
3D hexon structure models of Ad5, Ad5-37(5, 7) and Ad5-43(5, 7). These structure models were built using the Protein Modeling-Build Homology Models module in the Discovery Studio 2.5 software package. (**a**) Ad5 hexon trimer structures (Protein Data Bank 1P30) are shown using space-filling models (left panels) and ribbon diagrams (right panels). HVR1, red; HVR2, cyan; HVR3, yellow; HVR4, light green; HVR5, green; HVR6, orange; HVR7, blue. The remainder of the hexon trimer is shown in grey. (**b**) Analysis of possible intermolecular interactions between HVR1, HVR5 and HVR7 from another monomer. For 5H-37(5, 7), the extra HVR1 region points inward and forms a compact structure with HVR5 and HVR7. Two possible salt bridges exist, one between E154 in HVR1 and K423 in HVR7, and the other between D436 in HVR7 and K181 in HVR3, which is not far away from HVR5. For 5H-43(5, 7), an extra salt bridge is located between E137 in HVR1 and K429 in HVR7.

Several crystallography structures (3YIN, 1P3O) of Ad5 hexon are present in the Protein Data Bank (PDB), but none of them shows the structure of HVR 1, which likely indicates that some inherent structure of this region is inherently not stable^13^,^15^,^21^,^22^,^23^. Thus, we modeled the HVRs with reasonable geometry with respect to the chimeric hexon proteins and produced ribbon and space-filling diagrams. Our Ad5 hexon model with the extra HVR1 region directed outward is shown in Fig. 3a. Analysis of the potential intermolecular interactions between HVR1, HVR5 and HVR7 from another monomer suggests that only a hydrogen bond is possible between Q157 in the HVR1 region and N457 in the HVR7 region of the other monomer (Fig. 3b). For the Ad5-37(5, 7) mutant, the extra HVR1 region points inward, thus forming a more compact structure with HVR5 and HVR7. Furthermore, two possible salt bridges exist, one between E154 in HVR1 and K423 in HVR7, and the other between D436 in HVR7 and K181 in HVR3, which is not far away from HVR5. For the Ad5-43(5, 7) mutant, the HVR1 region also points outward, but an extra salt bridge between E137 in HVR1 and K429 in HVR7 of the other monomer probably exists.

According to the sequence alignment, HVR1–6 is located in the region of the sequence (135–428) embracing loop 1, while HVR7 is located in loop 2. In the hexon trimer, the top of the subunit consists of loop 1, loop 2 and loop 4, each of which is on a different subunit and interacts intimately with the others. For example, loop 1 from one subunit is sandwiched between loop 4 of its immediate neighbor across the subunit interface and loop 2 from the third subunit. This region of the molecule is structurally significant. In addition, loop 4 has one long pair with more regular hydrogen bonding than the two short pairs in loop 2. This model suggests that loop 1 and loop 2 are in close contact with loop 4 to form the tower region of the hexon trimer (Fig. S5). Therefore, viral genomes including pAd5-37(1–7, L4), pAd5-43(1–7, L4), pAd5-37(loop 124) and pAd5-43(loop 124) were constructed and recombined as schematically shown in Fig. S6. Results showed that none of these vectors could be proved viable, and hexon trimers were not detected in Ad5-37(1–7, L4), Ad5-37(loop 124) or even Ad5-43 (loop 124) neither (Fig. S6). Together, these findings implied that modification of heterologous loops did not have a crucial effect on chimeric hexon polymerization, and the failure of chimeric hexon folding into trimers was not due to the stability of hexon intrasubunit interactions.

### L4-100K chaperone protein assists chimeric hexon in trimerization and nuclear location

**Figure 4:**
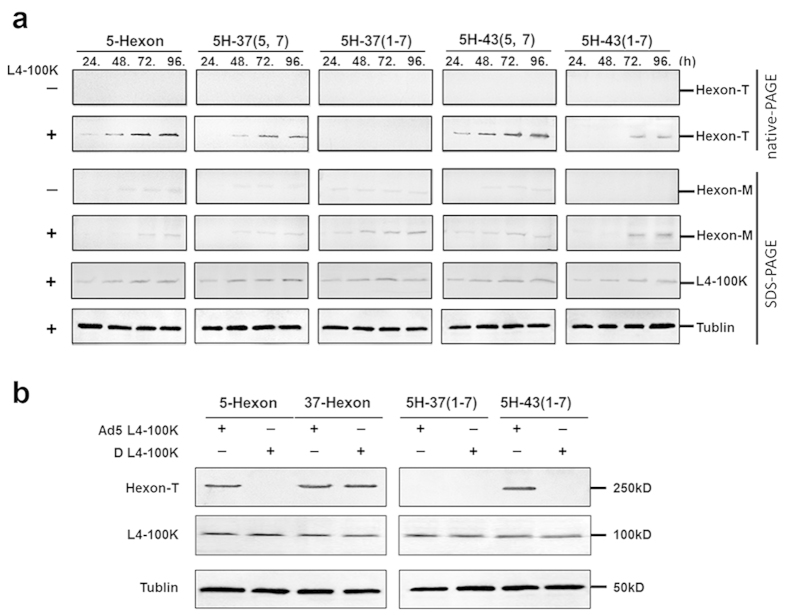
Chimeric hexon trimerization in presence of L4-100K protein. Chimeric hexon plasmids with or without the L4-100K plasmid were co-transfected into HEK293 cells, which were harvested at the indicated times points. (**a**) Total cell extracts were prepared, hexon trimers were separated by native-PAGE and subjected to immunoblotting using anti-hexon goat pAbs, hexon monomers were separated by SDS-PAGE and subjected to immunoblotting using anti-hexon goat pAbs, Ad5 L4-100K protein was detected by immunoblotting using an anti-HA mouse mAb. (**b**) Serotype-specific response between Ad5 L4-100K, subgroup D L4-100K and 5-Hexon plasmids were investigated by co-transfection into HEK293 cells as indicated, and Western blotting was performed at 72 h. All co-transfection experiments were repeated three times.

The assembly of hexon trimers is dependent upon the presence of a viral scaffold protein, the late phase non-structural protein L4-100K^24^,^25^,^26^,^27^,^28^. Previous studies have suggested that the L4-100K protein is required in stoichiometric amounts to assist in the proper folding and trimerization of hexon, as well as its transfer to the nucleus^27^,^29^. In order to further analyze the precise interaction and nuclear transfer, we studied the association between chimeric hexon and L4-100K protein by cloning the following hexon genes into a eukaryotic plasmid: 5H-37(5, 7), 5H-37(1–7), 5H-43(5, 7), 5H-43(1–7), and hexon from Ad5 (5-Hexon) as a control. We then checked the expression level of chimeric hexon in the absence or presence of L4-100K at indicated time points to 96 h after transfection. Western blotting analyses were performed under denaturing and non-denaturing conditions using anti-hexon goat pAbs from serotype 5. The results indicated that the expression levels of 5H-37(5, 7), 5H-37(1–7), 5H-43(5, 7) and 5H-43(1–7) monomers were similar to that of the wild-type in the presence of L4-100K and higher than that in the absence of L4-100K, correspondingly. However, the L4-100K protein was capable of assisting 5H-37(5, 7), 5H-43(5, 7) and 5H-43(1–7) proteins in trimerization, but not 5H-37(1–7) even at 96 h after co-transfection. Moreover, the 5H-43(5, 7) trimers were detected at 24 h after co-transfection, which was earlier and more efficient compared to those of 5-Hexon. Meanwhile, the 5H-43(1–7) trimers were detected at 72 h later than those of 5-Hexon (Fig. 4a). Next, an assay was developed to confirm the serotype-specificity between the L4-100K protein from Ad5 and hexon sequences from subgroup D. Accordingly, the expression plasmid for the L4-100K protein from subgroup D (D-100K) was constructed. Chimeric hexon plasmids, including 5H-37(1–7) and 5H-43(1–7), together with the D-100K plasmids were co-transfected into HEK293 cells, and Western blotting was performed after 72 h (Fig. 4b). We found that the Ad5 L4-100K protein could assist in the trimerization of 37-Hexon and 5H-43(1–7) proteins but not the 5H-37(1–7) protein. Interestingly, the D-100K protein could assist the trimerization of 37-Hexon but not 5H-43(1–7) nor 5H-37(1–7). These results indicated that the failure of 5H-37(1–7) to trimerize could not be attributed to the chaperonin L4-100K protein from Ad5, although serotype-specific epitopes are present in subgroup C and subgroup D.

**Figure 5:**
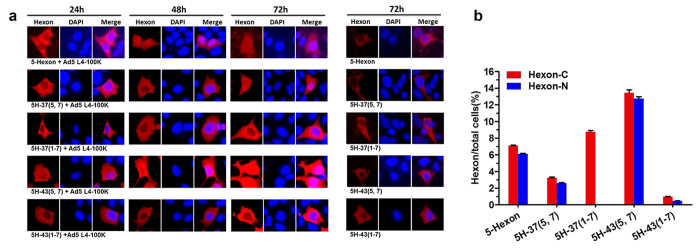
Nuclear transfer of chimeric hexons in presence of L4-100K protein. (**a**) Expression of variant hexons was observed in HEK293 cells transfected with 1 μg of each plasmid by confocal microscopy. At least 30 independent cells were examined in each sample, and the most representative cells are shown. Hexon-C: hexon in cytoplasm, Hexon-N: hexon transferred into nucleus. Blue: cell nucleus. Red: hexon protein. (**b**) The proportions of positive cells expressing hexon in the cytoplasm or transferred to the nucleus at 72 h post transfection were calculated.

Here, we focused on analyzing the relative importance of proper protein folding and the intracellular interaction of the L4-100K protein with chimeric hexons. Studies in recent years have shown that L4-100K behaves both as a cytoplasmic and nuclear protein, which means that both hexon monomers and trimers are present within the cytoplasm, whereas only hexon trimers are found in the nucleus^10^,^30^. To identify the nuclear translocation of chimeric hexon in the presence of Ad5 L4-100K, we detected intracellular protein localization by an indirect confocal immunofluorescence technique. HEK293 cells were co-transfected with 5-Hexon, 5H-37(5, 7), 5H-37(1–7), 5H-43(5, 7) and 5H-43(1–7) in the presence of Ad5 L4-100K. Cytoplasmic and nuclear protein localization was evaluated by an indirect immunofluorescence technique using hexon antibodies at indicated time points (Fig. 5a). The proportions of positive cells expressing hexon protein in the cytoplasm or nucleus were also calculated (Fig. 5b). As expected, all four chimeric hexon signals (in red) were already detected at 24 h in the cytoplasm, with increasing accumulation observed from 48 h to 72 h, and these expression levels were consistent with the Western blotting results above. Unlike 5H-37(5, 7) and 5H-43(5, 7), which generally were transported to the cell nucleus, no detectable signal for 5H-37(1–7) within the nucleus could be seen even after 72 h. Furthermore, the hexon fluorescence of 5H-37(1–7) observed in the cytoplasm was in the form of bright speckles, indicative of its inability to properly fold. By counting the number of fluorescent cells, we found no difference in the proportion of intranuclear hexon proteins among 5H, 5H-37(5, 7), 5H-43(5, 7) and 5H-43(1–7). These results suggested that this specific exchange of HVRs in hexon would affect its trimerization but not the nuclear transport of hexon trimers.

### Interaction between L4-100K protein and chimeric hexon

**Figure 6:**
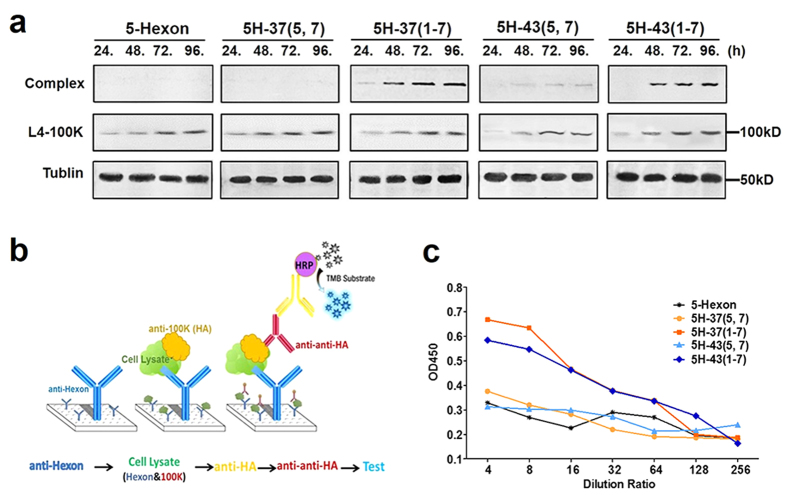
Binding analysis of chimeric hexon-100K protein complex. (**a**) 5-Hexon, 5H-37(1–7) and 5H-43(1–7), 5H-37(5, 7) and 5H-43(5, 7) were co-transfected with Ad5 L4-100K, and immunoblotting was performed by using an anti-hexon goat pAb, and Ad5 L4-100K was detected by using an anti-HA mouse mAb. (**b**) Schematic strategy for ELISA: immune plates were coated with an anti-hexon pAb and then incubated with cell lysates 72 h post transfection. HRP-conjugated anti-mouse secondary antibodies were used to detect positive signals. (**c**) OD450 values were detected for cell lysates at different dilution ratios. ELISA experiments were repeated three times.

To better understand the effect of L4-100K on chimeric hexon trimerization and transport, the interaction and binding region between L4-100K and hexon must be investigated. In earlier studies, stable complexes (~800kD) of the L4-100K protein with hexon monomers were shown to occur in adenovirus-infected cells^27^,^31^,^32^. Nascent hexon polypeptides would thus trimerize upon interaction with cytoplasmic L4-100K protein oligomeric complexes^17^. To identify the ability of the L4-100K protein to bind with chimeric hexon, Western blotting was performed as described above by using anti-hexon goat pAbs. We found that the hexon-L4-100K complex was somewhat different from that of hexon trimers (Fig. 6a). The 100K-Hexon complexes of 5H-37(1–7) and 5H-43(1–7) were observed to be more abundant than those of 5H-37(5, 7) and 5H-43(5, 7), with 5-Hexon used as a normal control. In order to confirm these results, an ELISA was designed as shown in Fig. 6b. We surmised that the L4-100K protein could be detected if 5H-37(1–7) and 5H-43(1–7) bound with the L4-100K protein when immune plates were coated with anti-hexon antibodies. The weak level of detection for the hexon-L4-100K protein complex with 5H-37(5, 7) and 5H-43(5, 7) could be a dissociation effect involving the proteolytic cleavage of the L4-100K protein, a phenomenon that we observed both in Western blotting and ELISA (Fig. 6c). The interaction process between hexon and L4-100K is accompanied by the precise displacement and timely liberation, so we could not detected the 800kD complex in Ad5 hexon. Our results suggest that the Ad5 L4-100K protein could recognize and bind 5H-37(1–7) monomers, but the trimerization or proper folding was inhibited.

**Figure 7:**
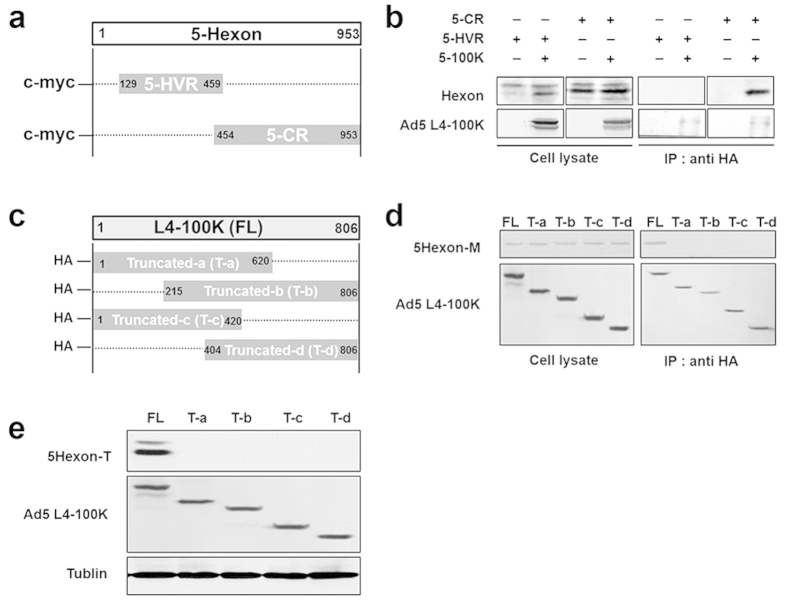
Mapping Ad5 L4-100K binding regions in chimeric hexons and effect of Ad5 L4-100K on hexon trimerization. (**a**) Schematic strategy to construct truncated hexons of 5-Hexon hypervariable region (5-HVR) and 5-Hexon conserved region (5-CR). (**b**) HEK293 cells (1 × 10^6^) were co-transfected with 2 μg HA-tagged Ad5 L4-100K and 2 μg 5-HVR or 5-CR. Cells were co-immunoprecipitated with HA beads followed by SDS-PAGE and immunoblot analysis using anti-HA and anti-hexon antibodies. (**c**) A schematic strategy to construct truncated Ad5 L4-100K. (**d**) HEK293 cells (1 × 10^6^) were co-transfected with 6 μg truncated L4-100K with HA-tag and 6 μg 5-Hexon. Cells were co-immunoprecipitated with HA beads followed by SDS-PAGE and immunoblot analysis using anti-HA and anti-hexon antibodies. All co-immunoprecipitation experiments were repeated three times. (**e**) HEK293 cells were transfected with Ad5 L4-100K, Ta, Tb, Tc, Td and 5-Hexon. Cells were harvested at 72 h. Total cell extracts were prepared, and hexon trimers were separated by native-PAGE and subjected to immunoblotting using an anti-hexon goat pAb and mouse tubulin mAb as a loading control.

All of these chimeric hexons were specifically replaced with the corresponding HVRs from Ad37 or Ad43. Thus, we did not alter the sequences of the conserved regions of hexon, as they form key elements for the secondary structure within the L4-100K protein and may be critical for hexon recognition and folding. Instead, we attempted to study this binding region by transfecting the conserved region of the truncated hexon (5-CR), HVR-truncated hexon (5-HVR) and HA-tagged L4-100K into HEK293 cells to perform an immunoprecipitation assay (Fig. 7a). Cell lysates were immunoprecipitated with HA beads, followed by SDS-PAGE and immunoblot analysis using anti-HA and anti-hexon antibodies. The experimental results showed that the L4-100K protein was capable of binding to 5-CR hexon proteins, but not with 5-HVR hexon (Fig. 7b). Two bands of hexon protein were detected, we considered the lower one as hexon protein. These data indicated that L4-100K could bind to the conserved region of hexon, and few 37 (1–7) and 43 (1–7) trimers were detected, not due to the failure of binding between hexon and 100K proteins, but rather to the inhibition of proper folding steps.

We also investigated the assistant region of the adenoviral L4-100K protein which plays a multi-functional role in virus assembly. According to earlier reports, we know that hexon binds to the L4-100K fragment from amino acid 215 to 420 and with lower affinity to the region from amino acid 614 to 806 *in vitro*^10^,^30^. In order to study the modification step of chimeric hexons in the presence of the L4-100K protein, we then constructed various truncations of L4-100K with a HA-tag as shown in Fig. 7c. These four truncated L4-100K proteins and 5-Hexon were co-transfected into HEK293 cells to perform an immunoprecipitation assay, and full-length L4-100K was used as a normal control (Fig. 7d). Cell lysates were immunoprecipitated with HA beads, followed by SDS-PAGE and immunoblot analysis using anti-HA and anti-hexon antibodies. The hexon trimers were also analyzed by native PAGE (Fig. 7e). Of note, the experimental results showed that the truncated L4-100K proteins were unable to bind to hexon proteins or to assist hexon folding into trimers effectively. These data indicated that L4-100K, as a chaperone protein, would serve in multiple functions of assisting in the proper folding and trimerization of hexon, as well as its transfer to the nucleus.

## Discussion

In this study, we generated several chimeric Ad5 vectors and unexpectedly found a special case which showed higher efficacy of the recombinant virus rescue and could be produced to higher titers than the rAd5 vector. This result indicated that some hexon-modified Ad5 vectors may decrease the cost of goods for production utilizing this technology. By comparison of hexon protein expression, we found that differences in the ability of chimeric hexon trimers to form may influence the viability and growth of HVR-exchanged rAd5 vectors.

Recent studies have reported the crystal and cryo-EM structures of the Ad5 capsid at 3.5-Å resolution, providing a detailed picture of the structural constraints of the hexon HVRs, including substantial contacts with minor capsid proteins^22^,^33^. The HVR loops residing on the surface of the virion interact with each other to stabilize the trimeric structure of the hexon^24^,^25^,^26^,^34^ and interact with neighboring hexon trimers or other capsid components^32^,^33^,^35^,^36^. Therefore, we first reasoned that a change in structural stability was introduced into the capsid of the hexon-modified vectors. Since the hexon trimers may determine the viability of the virus, we mainly focused on the stabilizing interactions in the hexon trimer. We therefore modeled the seven HVRs in the Ad5 hexon and established the homology models for the multi-HVR replacement hexons. We found no significant differences in hydrogen bonding, salt bridges and hydrophobic interactions, which are all considered to contribute to the stability of hexon trimers with exchanges either in HVR5, 7 or HVR1–7 (data not shown). X-ray crystallography of hexon has revealed a hexagonal “pedestal” base from which a “tower” region projects outward into the solvent. Three surface loops, loop 1, loop 2 and loop 4, from each monomer interdigitate to form the tower domain^10^,^14^,^37^,^38^. By constructing hexons with exchanges in HVR (1–7)-L4 and even loop 1, loop 2 and loop 4, however, we still could not improve the viability of chimeric Ad5 vectors and the expression of hexon trimers (Figs S5 and S6). These results indicated that the heterologous loops do not crucially affect chimeric hexon polymerization, although they are tightly interwoven.

Previous studies assumed that the inability to rescue hexon-exchanged Ad5 vectors is due to a lack of structural compatibility between the alternate hexon proteins and cognate proteins during the process of assembly (e.g., L4-100K scaffolding protein) and/or in the final capsid (e.g., penton, fiber, protein IX)^39^. However, Hong *et al.* demonstrated that the L4-100K protein of Ad2 could assist in the trimerization of subgroup C hexon and of subgroup B hexon, which implied that the functions of L4-100K are both homo- and heterotypic. In this study, we showed that the L4-100K protein from subgroup C was capable of assisting in folding 37-Hexon trimers correctly, although it was still unable to assist 37(1–7) trimerization (Fig. 4b). These results indicated the presence of other factors which could affect the hexon trimer formation. Thus far, several studies have reported the growth defect among chimeric adenovirus vectors^20^,^40^, and the authors posed the following reasons for these deficiencies in replication: 1) severe retardation of hexon folding into trimers which can delay the virus replication cycle; 2) antipathy of other major capsid proteins for the chimeric hexon, thereby causing stagnation of protein packing during virus assembly and production of progeny virus. However, the precise mechanism is still unclear. In our study, we also found a direct relationship between the hexon trimerization efficiency and yields of the chimeric adenovirus. For the growth defective vectors, Ad5-37(5, 7) and Ad5-43(1–7), the trimerization of these chimeric hexon proteins showed low efficiency. For the vector with growth improvement, Ad5-43(5, 7), its hexon trimerization had a relatively high efficiency (Figs 2 and 4a).

We therefore focused on the L4-100K protein that plays an important role in hexon folding mechanisms, which could limit viral assembly^10^,^17^,^30^,^32^. L4-100K as a molecular chaperone interacts with hexon proteins to assist in their maturation into trimers^22^,^27^,^30^,^41^,^42^,^43^. The binding of L4-100K proteins to the conserved region of hexon were demonstrated in Fig. 7e. In addition, the truncated L4-100K mutations could not assist hexon trimerization, full-length L4-100K was indispensable for hexon maturity (Fig. 7b,e). In our study, the truncated L4-100K proteins were unable to bind to hexon proteins, which were different from the early studies^10^. This might be related to possible differences in the expression system and/or length of truncated L4-100K mutations. We therefore speculated that the interaction between L4-100K and the nascent hexon may be mediated by the peptide binding domain rather than particular individual amino acids. This is a dynamic process that the substituted hexon HVRs may alter the spatial displacement of the L4-100K-hexon complex. Thus far, analyzing the L4-100K crystal structure is necessary to gain further insights into the role of the L4-100K protein during virus assembly. Altering the L4-100K protein would be another way to assist chimeric hexon maturation into trimers, especially in the HVR1–7 chimeric hexon substitutions, but studying such an aspect of the L4-100K would be complicated. With the onset of the late phase, L4-100K begins to perform a number of functions that are essential for efficient completion of the virus life cycle. L4-100K achieves these effects not only by acting as a chaperone for hexon trimerization, but also by contributing to the transport and selective translation of late viral mRNAs. Moreover, other factors assisting proteins and capsid components share the last 95 nucleotides of the L4-100K sequence, but using a different reading frame^10^,^44^. Moreover, the consensus amino acid sequence of the nuclear export signal (NES) between amino acids 383 and 392 implies a high conservation of this motif among different adenovirus serotypes, and the cytoplasmic localization of L4-100K may be critical for its multiple functions during replication. Therefore, modification of the L4-100K protein, as a regulatory protein, should be considered prudently.

**Figure 8:**
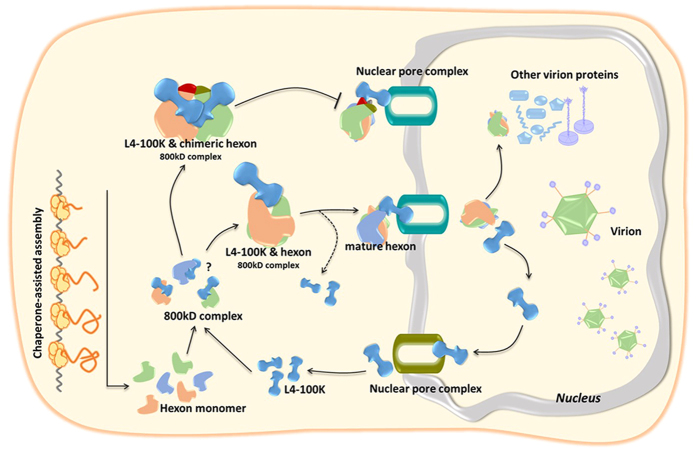
Schematic representation of mechanism of interaction between hexon and L4-100K. After transcription, nascent hexon polypeptide chains recognize L4-100K and bind to one side of this dumb-bell shaped protein. Once the hexon subunits bind with each other, a stable hexagonal base develops, and the L4-100K protein moves to the triangular top. The mature hexon then is transported into the nucleus by the chaperone. Some chimeric hexon subunits develop a stable hexagonal base as well as a stable triangular top, the function and the movement of the L4-100K protein become hindered.

Some plausible mechanisms that may explain the combined results observed with chimeric hexons in this study are illustrated in Fig. 8. After transcription, nascent hexon polypeptide chains can recognize L4-100K and bind to one side of this dumb-bell shaped protein. The interaction of hexon with the L4-100K protein has been shown to occur via one of the globular domains of the L4-100K protein molecule^30^. Once the hexon subunits form salt bridges and hydrogen bonds with each other, a remarkably stable hexagonal base develops. Meanwhile, the L4-100K protein moves to the triangular top, outward from the trimer surface. The mature hexon then is transported into the nucleus by the chaperone, and the interaction of L4-100K protein with hexon occurs in both cytoplasmic and nuclear compartments^10^,^30^,^45^. During the course of L4-100K protein nuclear export, hexon trimers together with other structural proteins assemble to form the capsid of progeny virions. As a regulatory protein, L4-100K plays a vital role in the late phase of the adenovirus life cycle when vast amounts of structural proteins are synthesized and the viral genome is efficiently encapsidated in the nucleus. This process is accompanied by the precise displacement and timely liberation between hexon and L4-100K. Some chimeric hexon proteins, such as 5H-37(5, 7), tend to form extra salt bridges or hydrogen bonds among HVRs. Therefore, the L4-100K protein would be hindered functionally when the hexon subunits develop a stable hexagonal base as well as a stable triangular top. The unmatched cycles between L4-100K liberation and virus packing would lead to the growth defect of Ad5-37(5, 7).

In summary, we have generated several hexon-chimeric rAd5 vectors and observed higher recombinant efficiency and growth improvement with a HVR-exchanged Ad5 vector compared with the rAd5 vector, although most hexon-chimeric Ad5 vectors constructed by us and other groups proved to be nonviable or have growth defects. Our structural and biochemical analyses suggest that the intricate interaction between hexon and L4-100K would determine the virus rescue and proliferation efficiency of hexon-chimeric rAd5 vectors. Further structural and functional studies on L4-100K will help to clarify the assembly mechanism of hexon-modified Ad5 vectors.

## Methods

### Cells and transfections

The human embryonic kidney cell line (HEK293) was obtained from the American Type Culture Collection (ATCC, USA) and grown in Dulbecco’s modified Eagle’s medium (DMEM) containing 10% fetal bovine serum (FBS; Hyclone, USA). Cells were cultured in a 5% CO_2_ atmosphere at 37 °C. Transfections of HEK293 cells were performed using Lipofectamine 2000 (Invitrogen, USA) according to the manufacturer’s instructions.

### Antibodies

The following antibodies were used in the present study: anti-tubulin mouse mAb (Covance, USA), anti-HA mouse mAb (Covance), anti-c-myc mouse mAb (Millipore, USA), anti-hexon goat pAb (Thermo Scientific, USA), anti-Ad5 pAb (Abcam, UK). The anti-Ad5 and anti-Ad37 rabbit polyclonal serum were a gift from Panyong Mao (Beijing 302 Hospital, Beijing, China). The anti-Ad5 L4-100K pAb was a gift from Patrick Hearing (Stony Brook University, New York, USA). The primary anti-penton and anti-fiber rabbit polyclonal serum (prepared by our laboratory) was used at a 1/100 dilution. Secondary antibodies were alkaline phosphatase-conjugated anti-rabbit, anti-mouse, rabbit anti-goat IgG or horseradish peroxidase-conjugated anti-mouse IgG (Jackson Immunoresearch, USA).

### HVR-chimeric Ad5 vectors

HVR-chimeric Ad5 vectors were generated by using a two-plasmid rescue method as previously described^18^. These recombination plasmids were designated as pAd5, pAd5-37(5, 7), pAd5-37(1–7), pAd5-43(5, 7) and pAd5-43(1–7) vectors (Fig. 1a).

### Identification of recombinant HVR-chimeric Ad5

The HVR-chimeric Ad5 genome plasmids together with the small plasmid pDC316-GFP expressing green fluorescent protein (GFP) under control of a mouse CMV promoter were co-transfected into HEK293 cells, yielding homologous recombinant vectors Ad5, Ad5-37(5, 7), Ad5-37(1–7), Ad5-43(5, 7), Ad5-43(1–7) vectors and Ad5-37(1–7, L4), Ad5-43(1–7, L4), Ad5-37(loop 124) and Ad5-43(loop 124). By selecting green fluorescent plaques, a single clone was isolated as described previously^46^, and then numbers of cells expressing the GFP transgene were counted. The HVR-chimeric rAd5 vectors were identified by PCR using the E2B genes and unique genes.

### Viral titers

The infectious titers (IFU/ml) of the HVR-chimeric rAd5 vectors were detected by a rapid titer method using HEK293 cells. A total of 2.5 × 10^5^ HEK293 cells were cultured per well in 24-well plates. After 2 hours, HVR-chimeric rAd5 samples were 10-fold serially diluted from 10^–2^ to 10^–6^ ml in DMEM. One hundred microliters of each viral dilution was added dropwise per well. Infected cell cultures were incubated at 37 °C in 5% CO_2_ for 48 h before fixing the cells by adding 500 μl of ice-cold 100% methanol to each well very gently. After the cells were incubated at −20 °C for 10 min, the methanol was aspirated, and the plates were rinsed three times with 500 μl PBS containing 1% BSA. The anti-Ad5 pAb was added at 1:1,000 dilution in PBS containing 1% BSA. The final rinse was aspirated from the wells, and then 250 μl of diluted anti-Ad5 antibody was added to each well. Cells were incubated at 37 °C for 1 h, washed three times in PBS and then incubated with secondary HRP-conjugated antibodies for 1 h at 37 °C. After removing the secondary antibodies, the 500 μl of DAB working solution (Max Vision, China) was added per assay well at room temperature for 20 min. The DAB was aspirated, and then add 1 ml of PBS was added to each well. Three fields of brown/black positive cells (an ideal field should contain 5 to 50 positive cells) at minimum were counted using a microscope with a 20X objective, and infectious titers (IFU/ml) were calculated for each well (of a 24-well plate) as follows:


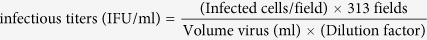


### Plasmid construction

Ad5 genes for HVRs of chimeric hexons [5-Hexon, 5H-37(5, 7), 5H-37(1–7), 5H-43(5, 7), 5H-43(1–7)], as well as those for L4-100K, Ad5-Hexon HVRs (5-HVR) and the Ad5-Hexon conserved region (5-CR) were generated from the appropriate viral genomic DNAs by PCR. All sequences were verified. Subsequently, the amplified fragments were cloned into the PDC316 vector (Novagen, USA) by using *Eco*R I/*Hin*d III restriction sites for eukaryotic expression. Two truncated Ad5-hexon (5-HVR, 5-CR) genes were generated from the hexon plasmids described above by PCR and then cloned into PDC316 by using *Sma* I/*Eco*R I restriction sites. A C-terminal c-myc tag was present in each of the plasmids.

### Western blotting analysis

Cells were collected at various time points post-infection or post-transfection and disrupted with lysis buffer (50 mM Tris, pH 7.5, with 150 mM NaCl, 1% Triton X-100 and complete protease inhibitor cocktail tablets). The samples were electrophoresed by SDS–PAGE or native-PAGE and transferred onto nitrocellulose membranes (Whatman, UK). SDS was not added into the transfer buffer (39 mM glycine, 48 mM Tris, 20% methanol, 1.3 mM SDS, pH = 9.2) when proteins were analyzed by native-PAGE. After blocking in 5% nonfat milk, the membranes were probed with various primary antibodies against proteins of interest. Secondary antibodies were used at a 1/3,000 dilution. Immunoreactions were performed with 0.1 M Tris (pH 9.5) containing 0.66% NBT solution and 0.33% BCIP solution.

### Immunofluorescence microscopy

The entry of hexon to the nucleus was evaluated by immunofluorescence. HEK293 cells (20–50% confluent) seeded on coverslips in a 24-well plate were transfected with plasmid DNA using Lipofectamine 2000. At various time points post-transfection, cells were fixed with 4% paraformaldehyde in PBS for 10 min at room temperature. For intracellular immunofluorescence, experiments were performed by permeabilizing the cells with 0.1% Triton X-100 for 8 min. Thereafter, the cells were washed three times in PBS, blocked with 10% FBS in PBS and then incubated with a goat anti-hexon pAb diluted 1:1,000 in PBS containing 1% FBS for 1 h at room temperature. Cells were washed three times in PBS and stained with Alexa Fluor 488 rabbit anti-goat IgG (Invitrogen) diluted 1:1,000 in PBS containing 1% FBS along with 1 mg/ml DAPI (4,6-diamidino-2-phenylindole) for 1 h at room temperature. The cells were then washed three times in PBS and incubated with ER-Tracker (Invitrogen) for 15 min at 37 °C. For surface immunofluorescence determinations, the same protocol was followed, except the Triton X-100 step was not performed. Stained cells were washed three times in PBS before detection. Fluorescent images were obtained using a Zeiss LSM710 confocal microscope equipped with a 40 × objective.

### Immunoprecipitation assay

At 72 h after transfection with indicated plasmids, HEK293 cells were obtained and dissociated in lysis buffer at 4 °C for 1 h, followed by centrifugation at 10,000 × *g* for 10 min at 4 °C to pellet the cell debris. The pre-cleared supernatants were collected and then mixed with anti-HA Ab-conjugated agarose beads (Roche, Germany), followed by incubation at 4 °C for 3 h. The beads were washed three times with wash buffer (20 mM Tris, pH 7.5, with 100 mM NaCl, 0.1 mM EDTA and 0.05% Tween 20), pelleted and then resuspended in 30 μl glycine HCl (pH 2.0) elution buffer. The eluted materials were subsequently analyzed by Western blotting.

### ELISA

At 72 h after transfection with indicated plasmids, HEK293 cells were obtained and dissociated in lysis buffer at 4 °C for 1 h, followed by centrifugation at 10,000 × *g* for 10 min at 4 °C to pellet the cell debris. The pre-cleared supernatants were collected and then diluted with PBST containing 2% BSA. For ELISA, flat-bottom Immune FEP-101 96-well plates (JET BIOFIL, China) were coated overnight with 0.1 μg/well of the anti-hexon pAb. Plates were washed three times in PBS containing 0.2% Tween20 (PBST), blocked with PBST containing 2% BSA and then incubated in triplicate with diluted cell lysate for 2 h at 37 °C. Plates were washed again and incubated with 1:1,000 anti-HA antibody for 2 h at 37 °C. Plates were washed again and incubated with 1:1,000 HRP-conjugated anti-mouse secondary antibodies. After the final wash, plates were developed with 100 μl of 3, 3′, 5, 5′-tetramethylbenzidine (TMB, QIAGEN, Germany) for 20 min at room temperature and stopped after 10 min by adding 50 μl of 2 M H_2_SO_4_.

### Statistical analysis

Statistical calculations were performed using the GraphPad Prism 5.0 statistical program. Statistical significance was determined by one way T-test with the P value < 0.05.

## Additional Information

**How to cite this article**: Yan, J. *et al.* Interaction between hexon and L4-100K determines virus rescue and growth of hexon-chimeric recombinant Ad5 vectors. *Sci. Rep.* **6**, 22464; doi: 10.1038/srep22464 (2016).

## Supplementary Material

Supplementary Information
