# Supplementary material for: Interaction between hexon and L4-100K determines virus rescue and growth of hexon-chimeric recombinant Ad5 vectors
Source: Sci Rep. 2016 Mar 3;6:22464. doi: 10.1038/srep22464 (PMC4776158; doi:10.1038/srep22464)
Supplement: Supplementary Information [file srep22464-s1.doc]

**Interaction between hexon and L4-100K determines virus rescue and growth of hexon-chimeric recombinant Ad5 vectors**

Jingyi Yan ^1, ¶^, Jianing Dong ^1, ¶^, Jiaxin Wu ^1^, Rui Zhu ^1^, Zhen Wang ^1^, Baoming Wang ^1^, Lizheng Wang ^1^, Zixuan Wang ^1^, Haihong Zhang ^1^, Hui Wu ^1^, Bin Yu ^1*^, Wei Kong ^1, 2^, Xianghui Yu ^1, 2, *^

**
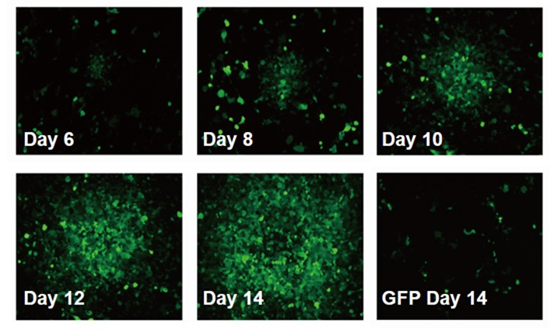
**

**Figure S1.** The HVR-chimeric Ad5 genome plasmids together with the shuttle plasmid expressing GFP were co-transfected into HEK293 cells (1×10^6^ cells in 10 cm^2^ dish). After indicated days of culture, single cytopathic effect (CPE) plaques expressing GFP were picked.

**
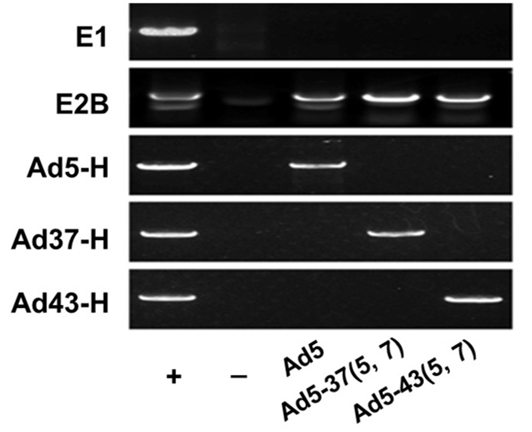
**

Figure S2. The HVR-chimeric Ad5 genome plasmids together with the shuttle plasmid expressing GFP were co-transfected into HEK293 cells (1×10^6^ cells in 10 cm^2^ dish). After indicated days of culture, single cytopathic effect (CPE) plaques expressing GFP were picked. After 14 days of culture, single clones were selected for generation and identification by PCR.

**
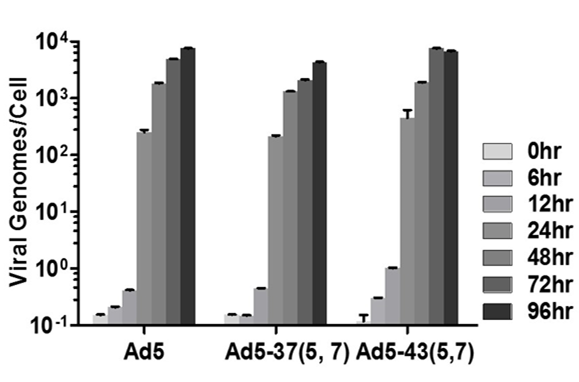
**

**Figure S3.** HEK293 cells were infected with the indicated virus at an MOI of 10 IFU/cell. Viral DNA was extracted at the indicated time points and analyzed by qPCR. Genome copy numbers per cell were calculated. Error bars indicate standard errors of the means (n=3).

**
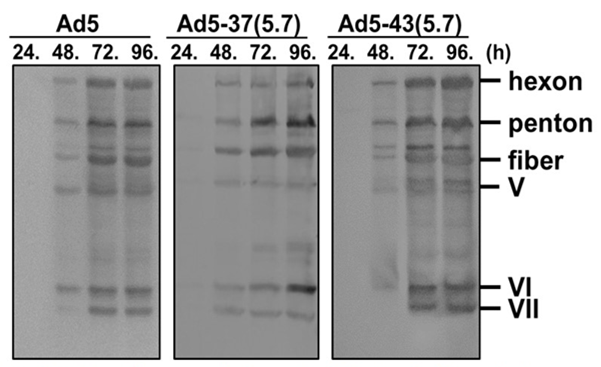
**

**Figure S4.** HEK293 cells were infected with the indicated virus at an MOI of 10 IFU/cell. To assess late adenovirus proteins accumulation, infected cells were obtained, separated by SDS-PAGE and subjected to immunoblotting using anti-Ad5 polyclonal antibodies. All infection experiments were repeated three times.

**
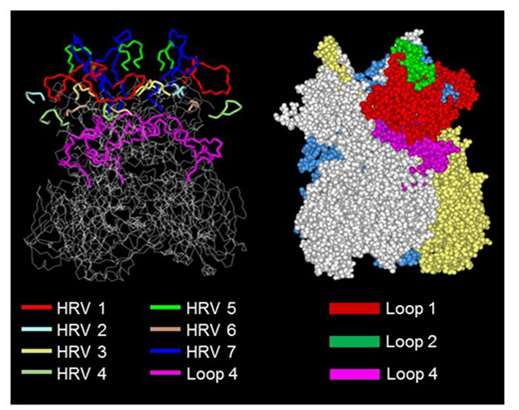
**

**Figure S5.** Ad5 hexon trimer structures (Protein Data Bank 1P30) are shown using space-filling models at the left side, HVR1, red; HVR2, cyan; HVR3, yellow; HVR4, light green; HVR5, green; HVR6, orange; HVR7, blue; loop 4, pink, ribbon diagrams at the right side, loop 1, red; loop 2, green; loop 4, pink. The remainder of the hexon trimer is shown in grey.

**
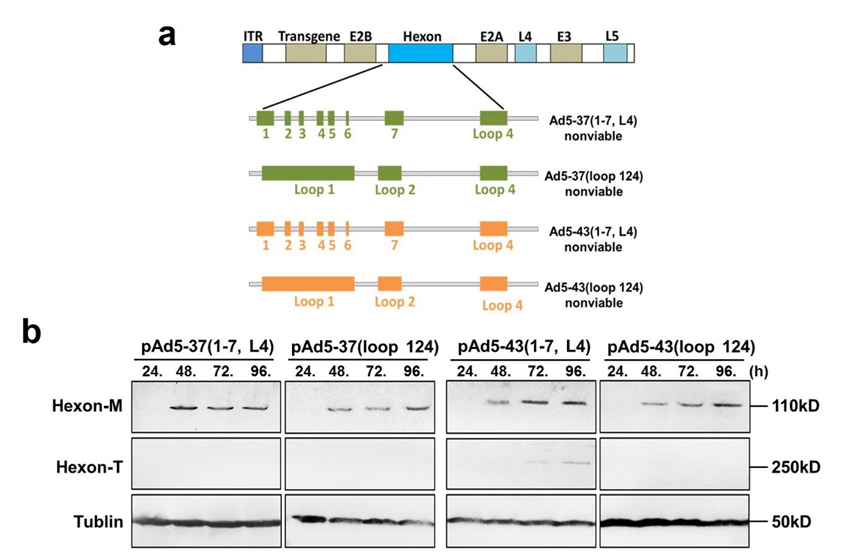
**

**Figure S6. (a)** A schematic strategy to construct Ad5-37(1–7, L4), Ad5-37(loop 124), Ad5-43(1–7, L4) and Ad5-43(loop 124) vectors by exchanging the HVRs and loop 4, or loop 1, loop 2, loop 4 with the corresponding regions. The Ad5 genes with the hexon protein, indicated in blue, are shown above. HVR sequences from Ad37 are indicated in green and Ad43 in orange. **(b)** HEK293 cells were transfected with pAd5-37(1–7, L4), pAd5-37(loop 124), pAd5-43(1–7, L4) and pAd5-43(loop 124) plasmids. Cells were harvested at the indicated time points. Total-cell extracts were prepared, and hexon trimers were separated by native-PAGE and subjected to immunoblotting using anti-hexon goat pAb from serotype 5 and mouse tubulin mAb as a loading control.

**Table 1.** Specific amino acid substitutions in the hexon protein of Ad5 in chimeric Ad5 vector backbones

|  | **Ad5-37(1–7)** | | **Ad5-37(5, 7)** | | **Ad5-43(1–7)** | | **Ad5-43(5, 7)** | |
| --- | --- | --- | --- | --- | --- | --- | --- | --- |
| Hexon ^a^ | Ad5 aa ^b^ deleted | Ad37 aa inserted | Ad5 aa deleted | Ad37 aa inserted | Ad5 aa deleted | Ad43 aa inserted | Ad5 aa deleted | Ad43 aa inserted |
| HVR1 | 136-165 | 136-157 |  |  | 136-165 | 136-159 |  |  |
| HVR2 | 188-194 | 174-184 |  |  | 188-194 | 176-186 |  |  |
| HVR3 | 212-220 | 202-209 |  |  | 212-220 | 204-211 |  |  |
| HVR4 | 248-258 | 237-249 |  |  | 248-258 | 239-251 |  |  |
| HVR5 | 268-281 | 258-264 | 268-281 | 258-264 | 268-281 | 261-273 | 268-281 | 261-273 |
| HVR6 | 305-310 | 288-293 |  |  | 305-310 | 297-302 |  |  |
| HVR7 | 418-451 | 401-438 | 418-451 | 401-438 | 418-451 | 410-440 | 418-451 | 410-440 |

^a^ Amino acid sequences of HVRs in hexon of Ad5, Ad37 and Ad43 were referred to sequences published by Roberts et al., 2006 ^1^.

^b^ aa, amino acids.

**Table 2.** Specific amino acid substitutions in the hexon protein of Ad5 in chimeric Ad5 vector backbones

|  | **Ad5-37(1–7, L4)** | | **Ad5-37(loop 124)** | | **Ad5-43(1–7, L4)** | | **Ad5-43(loop 124)** | |
| --- | --- | --- | --- | --- | --- | --- | --- | --- |
| Hexon | Ad5 aa deleted | Ad37 aa inserted | Ad5 aa deleted | Ad37 aa inserted | Ad5 aa deleted | Ad43 aa inserted | Ad5 aa deleted | Ad43 aa inserted |
| HVR 1-7 | a | a |  |  | a | a |  |  |
| loop 1 ^b^ |  |  | 132-320 | 132-303 |  |  | 132-320 | 132-312 |
| loop 2 ^b^ |  |  | 414-460 | 397-447 |  |  | 414-460 | 406-449 |
| loop 4 ^c^ | 801-858 | 788-845 | 801-858 | 788-845 | 801-858 | 790-847 | 801-858 | 790-847 |

^a^ The amino acid substitutions in the hexon of Ad5, Ad37 and Ad43 as well as those in Table 1.

^b^ Amino acid sequences of loop 1 and loop 2 in hexon of Ad5, Ad37 and Ad43 were referred to sequences published by Takeuchi et al., 1999 ^2^.

^c^ Amino acid sequences of loop 4 in hexon of Ad5, Ad37 and Ad43 were referred to sequences published by Roy et al., 1998 ^2,3^.

**Table 3.** The physical particles / infectious particles (VP/IFU) of the HVR-chimeric rAd5 vectors were detected.

| Adenovirus | Physical titer (VP/ml) | Infectious titer (IFU/ml) | Ratio (VP/IFU) |
| --- | --- | --- | --- |
| Ad5 | 8.8×10^12^ | 4.2×10^11^ | 21 |
| Ad5-37(5, 7) | 1.1×10^13^ | 3.2×10^10^ | 344 |
| Ad5-43(5, 7) | 2.8×10^13^ | 4.0×10^12^ | 7 |

**Reference**

1 Roberts, D. M. *et al.* Hexon-chimaeric adenovirus serotype 5 vectors circumvent pre-existing anti-vector immunity. *Nature* **441**, 239-243, doi:10.1038/nature04721 (2006).

2 Takeuchi, S., Itoh, N., Uchio, E., Aoki, K. & Ohno, S. Serotyping of adenoviruses on conjunctival scrapings by PCR and sequence analysis. *J Clin Microbiol* **37**, 1839-1845 (1999).

3 Roy, S., Shirley, P. S., McClelland, A. & Kaleko, M. Circumvention of immunity to the adenovirus major coat protein hexon. *J Virol* **72**, 6875-6879 (1998).
